# Public perception of Morgellons disease: Lay media controversies and patient perspectives

**DOI:** 10.1016/j.jdin.2025.01.011

**Published:** 2025-02-27

**Authors:** Chenan A. Huang, Logan R. Smith, Steven R. Feldman

**Affiliations:** a Department of Dermatology, Center for Dermatology Research, Wake Forest University School of Medicine, Winston-Salem, North Carolina; b University of South Florida Morsani College of Medicine, Tampa, Florida; c Department of Dermatology, Wake Forest University School of Medicine, Winston-Salem, North Carolina; d Department of Pathology, Wake Forest University School of Medicine, Winston-Salem, North Carolina; e Department of Social Sciences and Health Policy, Wake Forest University School of Medicine, Winston-Salem, North Carolina

**Keywords:** controversy, delusional infestation, delusional parasitosis, Morgellons disease, patient education, patient rapport, popular culture

*To the Editor:* Morgellons disease is a subtype of the broader diagnosis of delusional infestation.^1^ Described in 1674 by Sir Thomas Browne and later commandeered by Mary Leitao, Morgellons disease specifically refers to patients perceiving fibers embedded within the dermis, as opposed to parasites as in delusional parasitosis.^1^^,^^2^ A 2019 documentary, “Skin Deep: The Battle Over Morgellons,” further publicized Morgellons and criticized the assumption of a neuropsychiatric etiology. Controversy persists despite a Centers for Disease Control and Prevention study that determined a neuropsychiatric etiology through evidence of fibers consistent with skin protein and cotton from scanning electron microscopy, x-ray spectroscopy, and infrared spectroscopy.^2^^,^^3^ Leitao and the media’s popularization of Morgellons gave patients a shared identity, and many turned to Wikipedia to learn more. We analyzed the Wikipedia process underlying the Morgellons Wikipedia page to better understand patients’ perspectives and the quality of online content available to patients.

In the Wikipedia community, controversy on the Morgellons webpage began in August 2006 and continues to the present day (August 2024).^4^^,^^5^ Many contributors are self-diagnosed patients who wanted to build representation and community through Wikipedia. Contributors gave unfiltered perspectives, reporting successful treatment of close contacts and themselves with antibiotic, antiparasitic, and antifungal medications (Table I). Most common were extended courses of tetracycline, oxytetracycline (approved for use in animals only), and ivermectin. The leading alternative hypothesis is infection by *Borrelia* spirochetes, suggesting Morgellons is another manifestation of Lyme disease.^2^^,^^4^^,^^5^ Coinfection by the cellulose-producing *Agrobacterium* was suggested to explain the embedded fibers.^5^ Interestingly, although other theories regarding the etiology of Morgellons (nanobots, biologic warfare, and dental-amalgam psychosis) were mentioned in the discussion, these gained little traction.^5^ Insight to patients’ theories about their disease offers opportunities for clinicians to broach a sensitive topic.

**Table I:** Excerpts of discourse from the community talk page of the Morgellons Wikipedia page

Summary	Excerpts from the Morgellons Wikipedia talk page
August 2006, February 2007, and July 2013: Three different anonymous users describe their experience with Morgellons.	I have had the symptoms of Morgellons for four years. I thought I was totally alone. I just found out there is a name for it 2 weeks ago. It is not an internet hoax. It is very real and absolutely horrendous. I have been to 9 doctors and not one of them has taken it seriously. The scientist associated with the Morgellon website has received dozens of samples from different patients. They are all similar. He indicates in his interview video that the medical profession is ignoring something that is very real. August 25, 2006 (UTC) After 20 doctors, I finally was diagnosed with Morgellins from a parasitic specialist. I was successfully treated by Ivermectin 18 mg once a week for 6 weeks. The entire household had to be treated. […] During the 18 months my family had this - we infected @ 15 people/families. This is a parasite, the fibers are egg sacks. Oil or ointment, disolves the “glue” which holds these fibers under the skin, so when applied, in @ 10 minutes these fibers pop up & out of the skin. […] Acticin or Premrethryn is also effective over the entire body & scalp for 8 hours, once a week for 6 weeks. February 19, 2007 (UTC) […] A mainstream practicing dermatologist has diagnosed me with Morgellons and I have received multiple antibiotic treatments for it then why do you allow the propaganda that it is delusional to be portrayed as fact? I have seen VA psychiatrist in regards to this and they have confirmed that it is a real disease related to syphilis so it does have cognitive and memory loss aspects to it. July 5, 2013 (UTC)
October 2006: **User Pez** argues for balanced representation of all theories. **User Herd of Swine** argues that balanced representation does not equate to equal representation. Minority views are already represented as a minority proportion of the article.	**Pez**: The article also quotes doctors who say that the disease isn’t real. To be balanced, I think that it needs to quote doctors who do believe that the disease is real. October 28, 2006 (UTC) **Herd of Swine**: Pez, Wikipedia strives for a neutral point of view. Your point of view is that Morgellons is a real disease. Almost the entire medical community (all except a handful of doctors) thinks that Morgellons is catch-all mixture of symptoms and conditions that has been given a name for no good reason. The article should not reflect your point of view - it should reflect the known and accpted facts of the matter. This can include a description of the disagreements, but must be given appropiate perspective. All of dermatology vs a professor of physiology and a professor of biochemity is not a balanced debate. These are renegade and unorthodox scientists, bordering on pseudoscience. October 28, 2006 (UTC)
March 2007: **User RamyB777** assumes the CDC validated and accepted Morgellons as a disease with a known etiology. **User Dyanega** corrects RamyB777 and explains the difference between recognizing and validating Morgellons.	**RamyB777**: APPARENTLY, the CDC DID FIND MRF CREDIBLE and included them on their list as a reference for the rare disease. The CDC did not make a mistake. You are not higher than the governmental authority issuing the credence for the disease. March 16, 2007 (UTC) **Dyanega**: The CDC has not yet declared that this is a disease; the investigation has not yet produced a report. You, like Lakinekaki, seem not to understand that a person compiling a listing of known diseases is NOT actually evaluating the validity of the claims themselves. If, for example, the citation that caused Morgellons to appear in the CDC's listing is the publication by Savely, *et al*., then the process is circular; MRF board members publish a claim that Morgellons is a disease, and the CDC lists their publication, therefore MRF supporters insist that the CDC has validated their claims? In what way is that logical? That is most definitely NOT how it works. March 16, 2007 (UTC)
February 2013: **User BarclayAllan** adds Middelveen’s publications to the main Morgellons page of Wikipedia without consent from other users. **User Yobol** references Wikipedia’s internal standards for citations for why he removed BarclayAllan’s original addition.	**BarclayAllan**: I have just tried to add information about interesting new research by middelveen and others that has been published in peer-reviewed journals. Disappointingly, this was removed within minutes by “yobol”. Could yobol explain why peer-reviewed literature doesn't belong in a wikipedia article? February 11, 2013 (UTC) **Yobol**: Please review WP:MEDRS. Medical information needs to be sourced according to that guideline, specifically to high quality secondary sources such as review articles. The sources you presented are primary research articles, and do not qualify. Please also read WP:WEIGHT (in this case, the position that Morgellons is infectious in origin is an extreme minority opinion), and WP:PSTS, which also suggests secondary sources. February 11, 2013 (UTC)
June 2013: **User Drgao** cites numerous publications in support of the infectious etiology of Morgellons and argues for their inclusion on the main Morgellons page. **User Zad*68*** agrees that the number of sources do lend some credibility to an infectious etiology. However, the quality of each source is low and does not meet Wikipedia’s internal standards.	**Drgao**: In spite of this abundance of studies supporting the association of Morgellons with a spirochete infection of the skin, **several editors on this Wikipedia page have formed a consensus against including these studies on an infectious etiology of Morgellons, for no justifiable reason**. These editors prefer to maintain a biased point of view, which suppresses information about the infectious microbial associations of Morgellons, and seeks to present Morgellons as a purely psychological condition in which the skin lesions are not genuine (but self-inflicted by the patients — a ridiculous notion!). These editors are blatantly flouting the neutral point of view policy of WIkipedia (WP:NPOV). The above-cited studies MUST be referenced in this Wikipedia article, to ensure that all the material on Morgellons is covered in the article. Failure to include these studies flouts the neutral point of view policy of WIkipedia. These flouting editors always try to give some excuse for not including these above-cited studies, but their arguments and excuses are not valid. June 19, 2013 (UTC) **Zad*68***: Drgao, I agree that those are probably the strongest sources available for the microbe theory, and they get close to, but do not quite reach, the threshold for inclusion on Wikipedia. They're primary research, opinion pieces, a letter to the editor, not PUBMED indexed, not MEDLINE indexed, or published in journals that don't quite meet Wikipedia's cutoff. I think that that this research is close to meeting Wikipedia's requirements but - as others have noted here - it isn't quite there yet. I'd bet that within the next 2-3 years there will be satisfactory sourcing that will merit inclusion here, but it's just not quite there yet. June 19, 2013 (UTC)

These excerpts represent anecdotes and discussions that are not published on the main Morgellons Wikipedia page. Users (bold) discuss potential changes to the main page and reach consensus before altering the main Morgellons page. Although none of these excerpts involve intervention by a moderator, unresolved discussions may undergo arbitration before the main Wikipedia page is updated. The excerpts are chronological, and typos are unaltered from source material. Names, length, and layout are edited for clarity.

*CDC*, Centers for Disease and Prevention; *MRF*, Morgellons Research Foundation; *UTC*, coordinated universal time; *VA*, veterans affairs; *WP:MEDRS*, Wikipedia: identifying reliable sources (medicine); *WP:NPOV*, Wikipedia: neutral point of view; *WP:PSTS*, Wikipedia: primary, secondary and tertiary sources; *WP:WEIGHT*, Wikipedia: due and undue weight.

The spirochete theory is supported by the Morgellons Research Foundation, which was established by Mary Leitao to advance Morgellons research. Marianne Middelveen is frequently cited in Wikipedia discussions to support the spirochete etiology.^2^ Several Wikipedia contributors consistently removed Middelveen’s sources from the Wikipedia article, pointing out Middelveen’s questionable peer review and numerous self-references. The few outside citations to Middelveen’s work disagreed with the infectious etiology of Morgellons.^5^ To support Middelveen’s inclusion, Morgellons supporters argued that there should be *equal* representation of infectious and psychiatric etiologies of the disease.^5^ However, Wikipedia does not give equal representation to all views and instead favors a representative view. The minority, no matter how vocal, will be consistently represented as a minority on Wikipedia (Table I). The goal of Wikipedia is to remain behind the curve, maintaining its reputation as a tertiary source.

Wikipedia’s robust consensus review improved its reputation as a reliable online source, and its Morgellons discussion page can serve as a transparent reference. Wikipedia may serve as an intermediary for patients wary of conventional scientific platforms. The unusual organization of the discussion page addresses questions in a format that may connect well with patients. Especially for patients with Morgellons who may have distrust in traditional medical literature, referencing nontraditional sources can increase their trust in the clinician.

## Conflicts of interest

Dr Feldman has received research, speaking, and/or consulting support from a variety of companies, including Galderma, GSK/Stiefel, Almirall, Leo Pharma, Boehringer Ingelheim, Mylan, Celgene, Pfizer, Valeant, AbbVie, Samsung, Janssen, Lilly, Menlo, Merck, Novartis, Regeneron, Sanofi, Novan, Qurient, National Biological Corporation, Caremark, Advance Medical, Sun Pharma, Suncare Research, Informa, UpToDate, and National Psoriasis Foundation and is the founder and majority owner of www.DrScore.com and the founder and part owner of Causa Research, a company dedicated to enhancing patients’ adherence to treatment. The other authors have no conflicts of interest to declare.
